# Urinary Tract Infection Caused by Burkholderia cenocepacia in a Newborn: A Case Report

**DOI:** 10.7759/cureus.110318

**Published:** 2026-06-05

**Authors:** Oumayma Hamdani, Abderrazak Saddari, Mohamad Lahmer, Fatima-zahra Joudar, Said Ezrari, Mostafa Elouennass, Adil Maleb

**Affiliations:** 1 Laboratory of Microbiology, Mohammed VI University Hospital, Microbiology Unit, Laboratory of Bioresources, Biotechnology, Ethnopharmacology and Health, Faculty of Medicine and Pharmacy of Oujda, Mohammed First University, Oujda, MAR; 2 Microbiology Unit, Laboratory of Bioresources, Biotechnology, Ethnopharmacology and Health, Faculty of Medicine and Pharmacy of Oujda, Mohammed First University, Oujda, MAR; 3 Bacteriology, Mohammed V Military Hospital, Rabat, MAR

**Keywords:** burkholderia cenocepacia, case report, newborn infections, urinary burkholderia cenocepacia infection, urinary tract infection

## Abstract

*Burkholderia cenocepacia* (*B. cenocepacia*) is a strictly aerobic, nonfermenting, Gram-negative bacillus belonging to the *Burkholderia cepacia* complex. It is the most virulent and pathogenic species within this group. It poses a therapeutic challenge due to its intrinsic resistance to several commonly used antibiotics and the emergence of acquired resistance. It mainly causes respiratory infections in immunocompromised patients, particularly those with cystic fibrosis. We report a rare case of urinary tract infection caused by *B. cenocepacia* in a neonate admitted to the neonatal intensive care unit for respiratory distress. Cytobacteriological examination of urine identified *B. cenocepacia*, with identification confirmed by MALDI-TOF (matrix-assisted laser desorption ionization-time-of-flight mass spectrometry). The isolate was susceptible only to sulfamethoxazole-trimethoprim. The clinical course was marked by the patient’s death before the initiation of targeted antibiotic therapy.

## Introduction

The *Burkholderia cepacia* complex (BCC) is a group comprising at least 20 different species of aerobic, nonfermenting, Gram-negative bacteria. *B. cenocepacia* is the most extensively studied species because of its higher pathogenicity and greater antibiotic resistance compared with other species within the complex [1,2]. It is a ubiquitous bacterium found in natural environments, including soil, water, sediments, and the rhizosphere. In hospital settings, it spreads through contaminated surfaces, medical equipment such as nebulizers and catheters, disinfectants, and tap water [3]. It is a major opportunistic pathogen responsible for potentially fatal nosocomial infections in immunocompromised patients. It is mainly associated with severe pulmonary involvement, particularly in individuals with cystic fibrosis. However, *B. cenocepacia* is rarely involved in extrapulmonary infections, such as urinary tract infections [4].

This report describes a rare case of urinary tract infection caused by *B. cenocepacia* in a neonate and compares it with the few documented cases reported in the literature.

## Case presentation

The patient was a female premature neonate born at 30 weeks of gestation, the second twin from a twin pregnancy. She was admitted to the neonatal unit for poor suckling, grunting, and hypotonia. Clinical examination revealed respiratory distress, with tachypnea, cyanosis of the lips and extremities, and signs of increased work of breathing. The Silverman-Andersen score [5] was 6/10. Pulmonary auscultation revealed crackles, suggestive of pulmonary edema. A complete blood workup, including blood culture, was performed and was normal, except for an elevated procalcitonin level of 1.07 ng/mL (Table 1).

**Table 1:** Results of blood laboratory tests.

Test	Results	Reference Values
White blood cell count	13,000/mm³	5,000-20,000/mm³
Hemoglobin level	15.9 g/dL	14-20 g/dL
C-reactive protein	2.83 mg/L	<5 mg/L
Procalcitonin	1.07 ng/mL	<0.1 ng/mL
Serum sodium	137 mmol/L	135-145 mmol/L
Serum potassium	5.1 mmol/L	3.5-5.0 mmol/L
Serum creatinine	6.24 mg/L	6-12 mg/L

**Figure 1:**
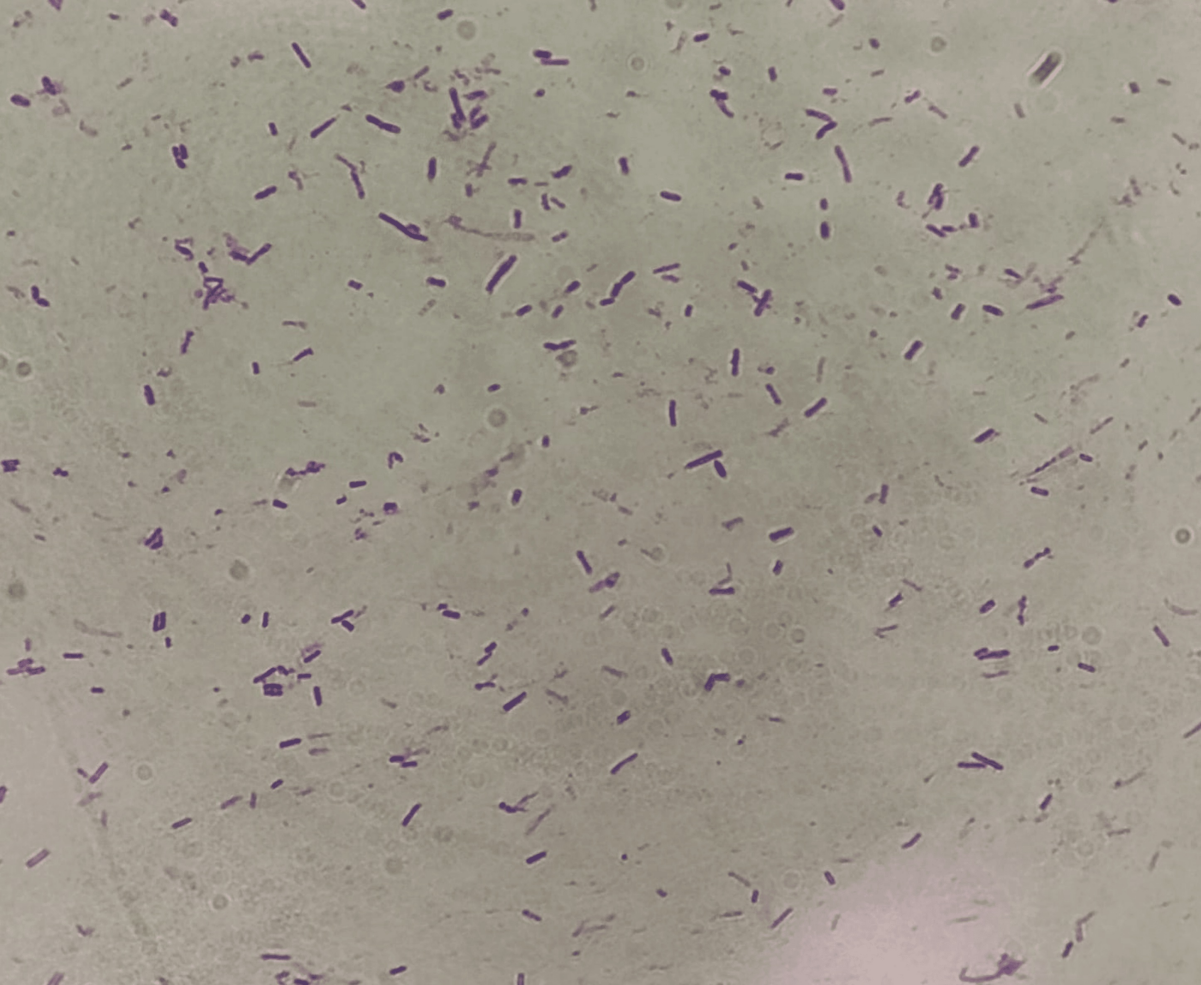
Burkholderia cenocepacia bacilli after Gram staining.

Cytobacteriological examination of the cerebrospinal fluid and transfontanellar ultrasound were also performed with normal results. A cytobacteriological examination of urine was requested, and the urine sample was collected using a sterile adhesive urine bag. On macroscopic examination, the urine was cloudy and yellow. Cytological analysis performed using the automated IRIS system showed positive leukocyturia, with 11,000 WBC/mL, without urinary crystals or casts. Gram staining showed pink, elongated, sometimes slightly curved Gram-negative bacilli, isolated or arranged in small clusters (Figure 1). Both catalase and oxidase tests were positive.

**Figure 2:**
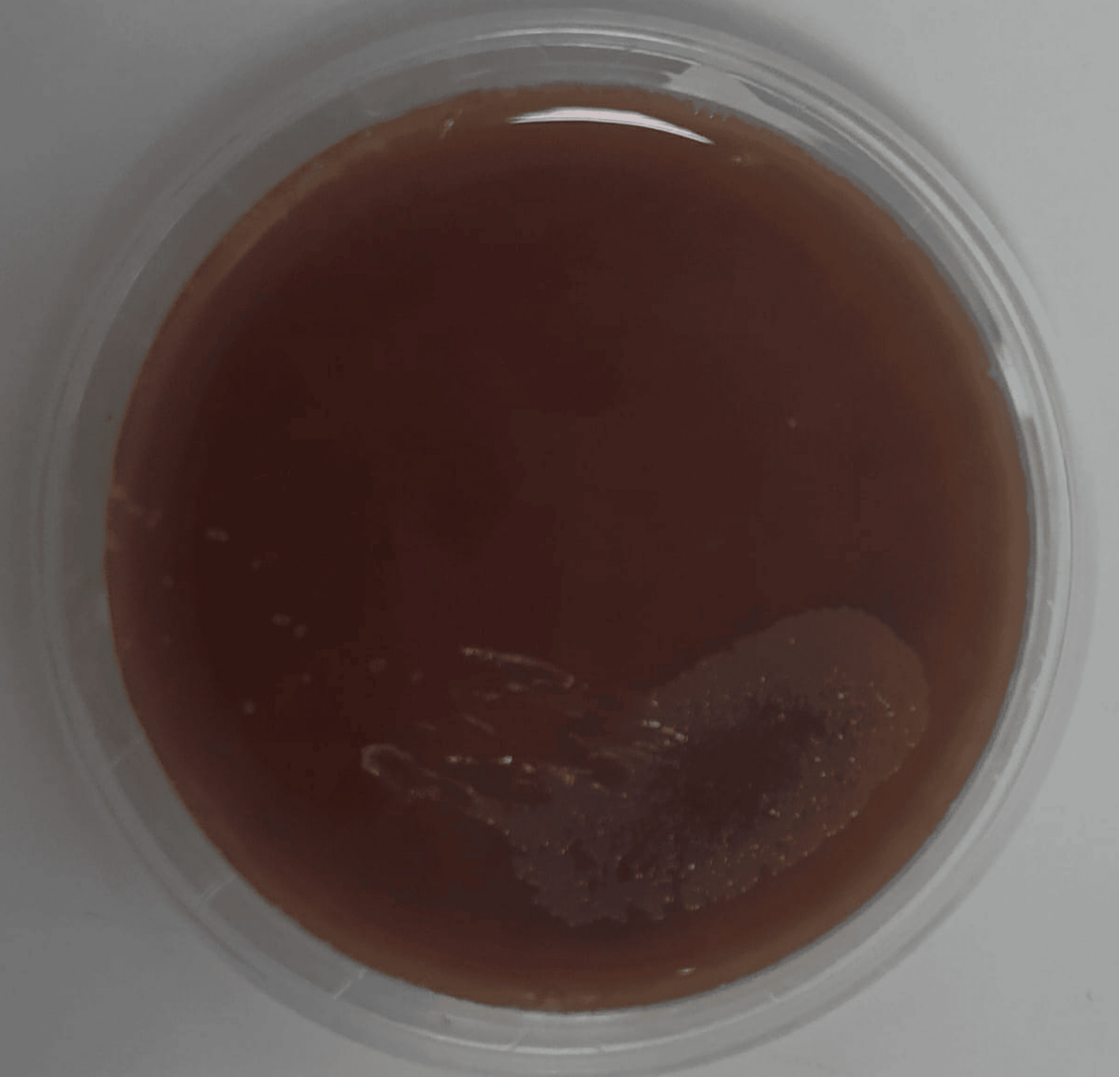
Burkholderia cenocepacia colonies on chocolate agar.

Identification of the organism was performed by MALDI-TOF mass spectrometry, confirming the species as *B. cenocepacia*. Culture on chocolate agar was positive after 24 hours of incubation, showing small, grayish, round, smooth colonies with regular borders, with a count of 10⁴ CFU/mL (Figure 2).

**Figure 3:**
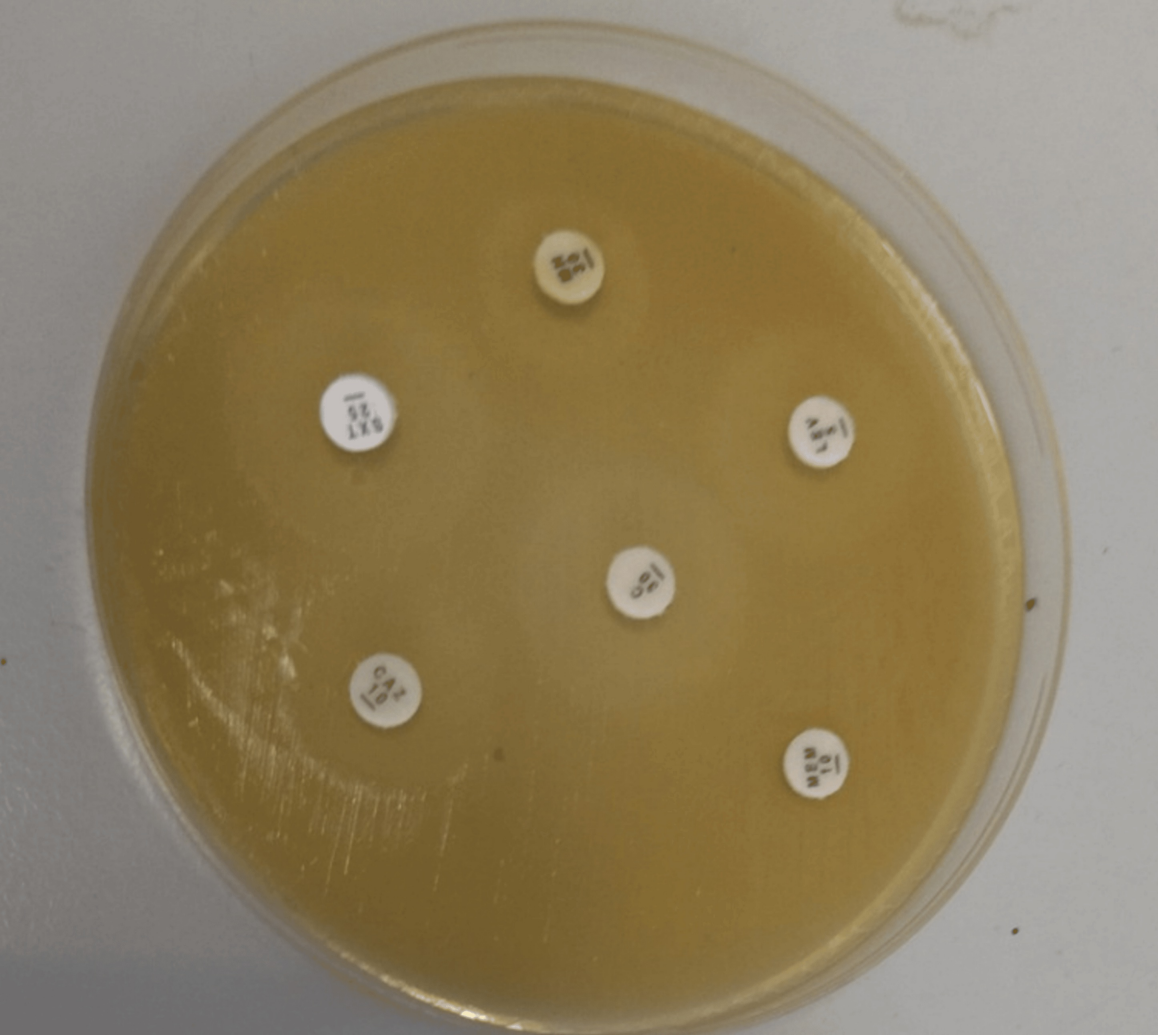
Antimicrobial susceptibility testing by the disk diffusion method on Mueller-Hinton agar.

Antimicrobial susceptibility testing by disk diffusion on Mueller-Hinton agar was performed according to the 2025 EUCAST guidelines [6] (European Committee on Antimicrobial Susceptibility Testing, Breakpoint Tables version 15.0, valid from January 1, 2025). The isolate was susceptible to sulfamethoxazole-trimethoprim, susceptible at increased exposure to ceftazidime and minocycline, and resistant to meropenem (Figure 3, Table 2).

**Table 2:** Antimicrobial susceptibility profile of Burkholderia cenocepacia isolate according to 2025 EUCAST guidelines.

Antibiotic tested	Interpretation
Sulfamethoxazole-trimethoprim	Susceptible
Ceftazidime	Susceptible at increased exposure
Minocycline	Susceptible at increased exposure
Meropenem	Resistant

Empirical antibiotic therapy with ampicillin and gentamicin was administered. The clinical course was unfavorable, with worsening respiratory distress, and the patient died in the context of severe neonatal sepsis.

## Discussion

The BCC bacterial complex, formerly known as *Pseudomonas cepacia*, consists of nonfermenting Gram-negative bacilli and was first identified in 1949. It represents a potentially pathogenic organism frequently encountered in nosocomial infections, particularly in immunocompromised patients, patients undergoing hemodialysis, and individuals with chronic pulmonary diseases such as cystic fibrosis [7]. *B. cenocepacia* is associated with mortality rates up to five times higher than those observed with other organisms within the BCC [8]. Urinary tract infections caused by *B. cenocepacia* are exceptional and have only occasionally been reported in the literature. However, they may pose significant diagnostic and therapeutic management challenges [9]. They often occur in patients with indwelling urinary catheters or after cystoscopy and are frequently associated with nosocomial outbreaks in settings requiring invasive care [10].

The identification of *B. cenocepacia* is complex because of its slow growth, usually requiring 48 to 72 hours, even on selective media such as BCSA (*Burkholderia cepacia* selective agar), PCA (*Pseudomonas cepacia* agar), and OFPBL (oxidation-fermentation polymyxin-bacitracin-lactose agar), as well as the phenotypic similarity among the different species. Commercial systems and MALDI-TOF may lead to inaccuracies in species-level identification. Therefore, molecular methods are recommended for reliable characterization, such as PCR (polymerase chain reaction) targeting the recombinase A gene, because isolated 16S rRNA gene sequencing is insufficient. The use of a combination of multiple markers, including gyrB (DNA gyrase subunit B), recombinase A, 16S rRNA, hisA (histidine biosynthesis gene), and rspU (30S ribosomal protein S21), as well as multilocus sequence typing (MLST), allows more accurate species identification and supports epidemiological investigations [8].

According to EUCAST guidelines, *B. cepacia* is intrinsically resistant to many antibiotic groups, including aminoglycosides, polymyxins, and several beta-lactams. In addition to its intrinsic resistance, this bacterium uses efflux pump mechanisms that actively expel antibiotics from the cell, reducing their intracellular concentration and, consequently, their therapeutic efficacy. Studies have shown that overactivation of certain efflux pumps, such as AmrAB-OprA, BpeAB-OprB, and BpeEF-OprC, in some *B. cenocepacia* isolates leads to specific resistance profiles [11,12].

Furthermore, the BCC is known to have the ability to form biofilms, which further reduces the effect of antibiotics. As a result, the therapeutic approach to infections caused by *B. cenocepacia* is particularly difficult because of these defense mechanisms [6,13]. This finding was observed in our patient, in whom the pathogen was susceptible only to sulfamethoxazole-trimethoprim. Our isolate showed resistance to meropenem, a beta-lactam antibiotic, which is consistent with the trend observed in other cases of *B. cepacia* infection, in which resistance to beta-lactams is common [14-16].

## Conclusions

The death of our patient highlights the clinical and diagnostic challenges associated with *B. cenocepacia*. The culture of this bacterium on standard media may fail. In our case, the use of MALDI-TOF mass spectrometry allowed identification of the isolate. This illustrates the important role of advanced diagnostic tools in detecting this organism in biological specimens. Rapid diagnosis is essential to initiate targeted therapy and optimize the chances of treatment success. Such infections may cause serious complications, particularly in patients with multiple risk factors, such as immunosuppression or the presence of invasive devices. Prompt management, careful monitoring, and the implementation of therapeutic strategies tailored to the specific characteristics of the identified organism are necessary to manage these infections effectively.
